# The nutrition transition in Malaysia; key drivers and recommendations for improved health outcomes

**DOI:** 10.1186/s40795-020-00348-5

**Published:** 2020-06-29

**Authors:** Ee Von Goh, Susan Azam-Ali, Fiona McCullough, Soma Roy Mitra

**Affiliations:** 1 Crops For the Future Research Centre (CFF), Semenyih, Malaysia; 2 School of Biosciences, Faculty of Science and Engineering, University of Nottingham Malaysia, Semenyih, Malaysia; 3 School of Biosciences, Faculty of Science, University of Nottingham United Kingdom, Nottingham, UK

## Abstract

**Background:**

The main purpose of this paper is to understand the multidimensional phenomenon of the nutrition transition in Malaysia, from 1980 to 2014, to inform future policies for a healthier nation.

**Methods:**

Food and health data were obtained through Food Balance Sheets, Malaysian Adult Nutrition Survey (MANS) and National Health and Morbidity Surveys (NHMS) for year-to-year review. Interaction between changes in food supplies and dietary trends and the progression of diet-related diseases and risk factors in tandem with demographic and socioeconomic transitions were observed using quasi-historical approach.

**Results:**

The period-under-review has seen Malaysia becoming more affluent, urbanised and modernised. Energy supply for Malaysian population remained consistently in excess of average calorie needs by a minimum of 30%. There were significant signs of shifting food trends, particularly in the supply of wheat (+ 56.5%), rice (− 23.7%), sugar and sweeteners (+ 23.9%), meat (+ 49.3%), fish and seafood (+ 38.7%), and eggs (+ 55.7%). The plant/animal protein ratio has decreased over time. Prevalence of NCD and associated risk factors has increased rapidly, some as high as 170%, despite various policy efforts to reduce them.

**Conclusion:**

The study highlights the importance of policymakers taking a relook into its policies and strategies, and formulate sustainable, comprehensive and multifaceted actions together with all relevant stakeholders to ensure a conducive, healthy and nutritious food systems and environment for its population.

## Background

The epidemiological transition, in particular the rapid shift in morbidity and mortality patterns towards much higher rates of non-communicable disease (NCD), has dominated the health profile of populations in higher income countries for the last half-century or more. Concurrent shifts in diet, activity levels and body composition also appear to be accelerating in many regions of the world [47]. Malaysia typifies a rapidly developing country that has undergone major demographic and socioeconomic changes since attaining independence in 1957. Transformation of the Malaysian economy from primary and secondary sector to tertiary sector has brought about rapid industrialisation and change of job patterns. Globalisation causes changes in the existing structure that bring the domestic economy closer to the international economy, mainly by improving absorption of labour, incomes and overall prosperity [55]. One of the expected impacts on dietary patterns relates to the globalisation of the food industry. As a result of direct foreign investment in food processing and retailing, and via global food advertising and local promotion, consumption patterns shift away from traditional local staples towards highly processed, often imported, food [18].

In recent decades, a rapid process of change in dietary trends coupled with a worsening obesity crisis has been observed in many developing countries [46]. Popkin [45] refers to these changes as “nutrition transitions”. In general, the transitions refer to the change from a traditional to a more ‘westernised’ or global diet and lifestyle, and from a largely agrarian based economy to an industrialised one. The changes are sequential and are distinguished by three distinctive phases – famine reduction, degenerative disease and behavioural change. This nutrition transition is preceded by changes in demographics, from populations with high rates of fertility and low life expectancy to lower fertility rates and longer life expectancy; and epidemiological transitions, from high rates of infectious disease, poor sanitation, periodic famine and malnutrition to a state where chronic disease and over-consumption prevail [44].

The nutrition transition is not new to Malaysia. Noor [41] reports on changes in eating habits and associated health outcomes since before the turn of the twenty-first century. Policymakers first recognised that the population was becoming overweight in the 1990s. To address this issue, strategies were outlined in the National Plan of Action for Nutrition in Malaysia (NPANM I) (1996 to 2000). The subsequent NPANM II (2006–2015) and NPANM III (2016–2025) recognised the needs to prevent and control diet-related NCD. As Malaysia being dubbed the fattest in Asia for the first time in 2014 [40] and continue to hold the record ever since, the strategies compel for a deeper look. To enable the development and implementation of effective policies for improved health outcomes, it is essential to understand the key drivers behind the nutrition transition in the country, and to assess which stage of the transition the nation is currently in. To achieve this, it is crucial to examine the nutrition transition in tandem with relevant policies that impact on food supply, diet and behaviour.

This paper focuses on the nutrition transition that has taken place in Malaysia over the last 30–40 years and highlights some important distinguishing features that have shaped the trend. We have chosen to adopt a quasi-historical approach to show how changes in food supply and dietary trends interact with the progression of NCD in roughly chronological order, using nationally representative data from multiple sources. To understand the demographic and epidemiological transitions, data are taken from the Department of Statistics, Malaysia (DoSM). Changes in food availability and dietary trends, are evaluated from the analysis of food balance sheets. Health data has been taken from the Malaysian Adult Nutrition Surveys (MANS) and the National Health and Morbidity Survey (NHMS). These interactions are used to gauge the effectiveness of the various national plans of action for nutrition in Malaysia (NPANM) in addressing the prevalence of non-communicable diseases (NCD).

To the best of the authors’ knowledge, this paper is the first one in Malaysia to appraise the change over time in Malaysian food and nutrition situation with policy implications.

## Methods

To evaluate the multidimensional phenomenon of the nutrition transition in Malaysia, literature searches were conducted from May to June 2017 and data were synthesised from multiple sources as detailed below.

### Demographic and socio-economic data

National demographics and socio-economic data were obtained from official spreadsheets and reports uploaded by the Department of Statistics, Malaysia (DoSM) on its open data portal [10]. These data included: (i) population growth rate, (ii) fertility rate, (iii) death rate and causes of death, (iv) under five mortality rate, (v) infant mortality rate, (vi) average life expectancy, (vii) old age dependency ratio, (viii) GDP index, (ix) household income, (x) broadband penetration rate, and (xi) vehicle ownership rate.

### Food and health data

To evaluate the Malaysian nutrition transition, data were gathered from three nationally representative sources, namely the Food Balance Sheets (FBS), Malaysian Adult Nutrition Survey (MANS) and National Health and Morbidity Survey (NHMS).

#### Food balance sheets (FBS) – 1980 to 2013

Food balance sheets (FBS) downloaded from FAOSTAT were used to assess food availability (in terms of kCal/capita/day) over the 34-year period from 1980 to 2013 [13]. To evaluate the trend in food supply over the past three decades, the years were clustered into subgroups with a range of 5 years for each group (i.e. 1980–1984, 1985–1989, 1990–1994, 1995–1999, 2000–2004, 2005–2009 and 2010–2013). The total energy available (kCal/capita/day) for the Malaysian population was estimated as the sum of energy supplied by animal and vegetable sources. The FAO definition of vegetable products includes the following: cereals, starchy roots, sugar crops, pulses, tree nuts, vegetable oil, vegetables, stimulants, spices, sugar and sweeteners, oil crops, fruits, alcoholic and miscellaneous; meanwhile, animal products consist of the following: meat, animal fats, eggs, milk (excluding butter), fish and seafood, aquatic product and offal [12].

Prior to 2003, when Malaysia carried out the first Malaysian Adult Nutrition Survey (MANS), FBS analysis was the most widely used approach to estimating food availability. Its biggest advantage is that the data is readily accessible and available online. FBS may be beneficial in showing the basic trends of food supply, but is not useful to assess the actual dietary intake of a population. FBS overestimated food consumption and nutrient intake compared to individual dietary surveys because FBS items were calculated excluding reuse and stock variation (national account budgets); they represented the total food items available per capita, but obviously not what was necessarily consumed. Despite the inherent inaccuracies of this method, food supply pattern is an important indicator of food consumption. An overabundance of food supply alone has been identified as a key cause of the obesity epidemic [54].

#### Malaysian Adult Nutrition Survey (MANS) - 2003 and 2014

In Malaysia, nationwide dietary intake data was collected for the first time in MANS 2003 and then subsequently in 2014. MANS was a nationwide cross-sectional study conducted on more than 7000 subjects. Multistage stratified sampling design was used to select a representative sample of the Malaysian adult population, aged 18 to 59 years old. Data on food consumption were derived from a Food Frequency Questionnaire (FFQ) that contains commonly consumed foods and beverages, and a 2 day 24-h diet recall. Primary data analysis reports for MANS 2003 and 2014 published by the Institute of Public Health were obtained from its official website [23–27, 29–31]. Information about food consumption pattern was extracted for comparison with the FBS data. Information about the prevalence of each weight status categories were then extracted and tabulated for period-to-period comparison.

Reliable nationally representative individual dietary surveys are crucial to better understand the relationship between food consumption patterns and the emergence of diet-related diseases. However, comparison of nutrient intakes is not possible because both the MANS showed that adult energy intakes were so low that they failed to meet at least 80% of the Malaysian RNI. With lower reported energy intake, the intake of many micro-nutrients was expected to decrease. It was pointed out in the report that there was a high percentage of under reporting during dietary recall, limitation in food composition database and human error during data management and analysis. After careful consideration of the high prevalence of underreporting, we have decided to report the trends of change in food consumption pattern between the two MANS surveys rather than the absolute value of change.

#### National Health and Morbidity Survey (NHMS) I (1986) to V (2015)

The National Health and Morbidity Survey (NHMS) is a nationally representative survey of the Malaysian population, from new-borns to the elderly. It was initiated in 1986 as a platform for monitoring the health of the Malaysian population. The interval of NHMS has been reduced from every 10 years to a 4 yearly cycle, with annual data collection since 2011. Since 2011, the main focus of the NHMS has been health care demands, non-communicable diseases and risk factors for NCD. Primary data analysis reports for NHMS III (2006), NHMS IV (2011) and NHMS V (2015) were downloaded from the Institute of Public Health [20, 28, 32]. The primary data analysis reports for NHMS I (1986) and II (1996) were not publicly available; hence, references were made to a PowerPoint presentation by Mr. A.J. Ahmad, Director of the Division of Health Promotion of the Ministry of Health (MoH) Malaysia, at a health promotion conference in 2011 [2]. Information about the prevalence of each weight status categories and NCD were then extracted and tabulated for period-to-period comparison.

## Empirical findings

### Transitioning demographic (DoSM)

The official statistics for the 34 year period indicate a downward trend in population growth rate coupled with an increase in life expectancy. During the 1991–2000 period, the rate of population growth was on average 3% per annum, decreasing to 1.8% during 2000–2010. Demographic forecasts expect this trend to continue with an estimated decrease in growth rate to 0.8% by 2040, an average decrease of population growth rate by 0.05% per year. This decline in population growth rate is a reflection of reduced fertility rate (from 6.19 births per woman in 1960 to 2.0 in 2015). Concurrently, Malaysia experienced a decline in annual death rate (from 8.17 per 1000 people in 1966 to 4.98 per 1000 people in 2015) and an increase in life expectancy. In 2016, average life expectancy was 77.2 years and 72.6 years for women and men respectively compared to 65.5 and 61.6 respectively for both genders in 1970. As a consequence, the Malaysia population is ageing. An increase in the old age dependency ratio, almost a three-fold increase from 7.4 (2010) to 21.7 (2040), is expected.

Malaysia is undergoing an epidemiological transition with causes of mortality shifting from communicable to NCD. Comparison of national statistics has shown that most deaths in Malaysia are now from NCD, with diseases of the circulatory system the most common cause of death. Nonetheless, the comprehensiveness of mortality data may be compromised since not all coders use International Classification of Diseases (ICD) codes [61].

### Rapid economic growth and transition (DoSM)

**Fig. 1:**
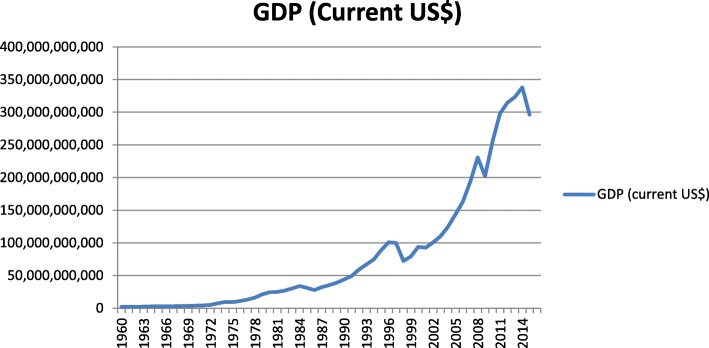
Malaysia GDP from 1960 to 2016 (Data adapted from DoSM [10])

Remarkable changes have occurred in the Malaysian economy and the structure of its workforce over the past 30–40 years. GDP per capita increased dramatically since the 1970s, achieving an average GDP growth rate of 6.8% per annum during the 1970–2015 periods (Fig. 1). With this rapid improvement in income, an increase in living standards is expected. A dramatic shift in urbanisation and modernisation of the population also occurred during this period. In 1960, approximately 27% of the Malaysian population was urban and 73% rural, a situation which totally reversed by 2015 (75% urban). Malaysia is transitioning from a largely agrarian economy to a service-oriented economy with tertiary sector accounting for 53.5% of GDP in 2014. While the economy has shifted from its initial dependence on an energy intensive workforce, during the 1987–2014 period, the rural primary-product sectors of agriculture, forestry and fisheries still accounted for 20% and manufacturing 23% of GDP in 2014.

In tandem with GDP growth, the Malaysian population experienced a rapid growth in household income. The annual growth of mean household income was about 11% in the 1990s and 9% annually since the 2000s up to 2014. During this period, the population became increasingly modernised and digitalised. Broadband penetration rate rose from 1.0% in 2004 to 72.2% in 2015. Vehicle ownership across all types also increased, with the percentage of Malaysian households owning cars as high as 83.9% in 2014. Increasing urbanisation has seen growing preference among Malaysians to spend their leisure time in shopping malls for food, social activities and entertainment. This habit has fuelled the mushrooming of shopping malls from just about 100 malls in the late 1980s to some 360 malls in 2015 [53], and close to 700 malls by the end of 2019 [33].

### Changes in trends of energy and nutrient supply (FBS review)

#### Energy supply

Over the 34 year period in review (1980–2013), the total energy supply (kCal/capita/day) for the Malaysian population was consistently in excess of average daily requirements. Based on the average daily requirements of 1500kCal and 2000kCal for women and men respectively [38], there was an excess of 75–93% available for women and 30–45% for men. The total energy per capita supply was stable over time with just a slight increase of about 5% over the last three decades (Table 1). However, there were rather significant signs of shifting trends in different food groups, in particular in the proportions of calories supplied by wheat, rice, sugar and sweeteners, poultry, fish and seafood, milk and eggs. While period-to-period variability (possibly due to external factors e.g. geopolitics and economic issue or seasonality) is observed, the long term trend is upward for energy supplied by wheat, sugar and sweeteners, poultry, fish and seafood and eggs and downward for rice and milk. The amount of energy per capita supplied by rice fell by 23.7% while that from wheat rose by 56.5% (Table 1). Available calories from sugar and sweetener increased by 23.9% over the past three decades (Table 1), from 21 to 26.1 tsp. per capita per day. Calories from animal products too increased (poultry by 230.7%; fish and seafood by 38.7%; egg by 55.7%). The only exception was milk, which decreased by 30% (Table 1).

**Table 1:** Daily per capita dietary energy supply (kCal) by products over the 34-year period (Source: Adapted from [13])

Year	Grand Total	Total Cereal	Wheat & Products	Rice	Sugar & Sweeteners	Total Animal Products	Red Meat & Offal	Poultry	Fish & Seafood	Eggs	Milk	Fruits (grams)	Vegetables (grams)
80–84	2758.0	1295.0	249.6	999.4	326.8	415.8	112.2	46.8	78.4	37.4	102.2	150.0	67.4
85–89	2638.2 (− 4.3%)	1081.8 (− 16.4%)	246.6 (− 1.2%)	785.2 (− 21.4%)	343.6 (+ 5.1%)	442.0 (+ 6.3%)	122.0 (+ 8.7%)	65.6 (+ 40.2%)	78.8 (+ 0.5%)	42.0 (+ 12.3%)	97.8 (−4.3%)	150.0 (0.0%)	65.2 (−3.1%)
90–94	2739.4 (+ 3.8%)	1158.0 (+ 7.0%)	269.6 (+ 9.3%)	826.8 (+ 5.3%)	374.6 (+ 9.0%)	538.6 (+ 21.9%)	160.0 (+ 31.1%)	100.0 (+ 52.4%)	86.0 (+ 9.1%)	51.2 (+ 21.9%)	107.8 (+ 10.2%)	144.0 (− 3.9%)	76.4 (+ 17.3%)
95–99	2909.2 (+ 6.2%)	1251.2 (+ 8.0%)	289.2 (+ 7.3%)	848.6 (+ 2.6%)	469.2 (+ 25.3%)	563.0 (+ 4.5%)	150.6 (−5.9%)	120.8 (+ 20.8%)	103.0 (+ 19.8%)	50.6 (−1.2%)	107.8 0%	145.4 (+ 0.8%)	95.0 (+ 24.2%)
00–04	2811.2 (−3.4%)	1255.4 (+ 0.3%)	346.6 (+ 19.8%)	752.8 (−11.3%)	381.2 (− 18.8%)	521.4 (−7.4%)	114.0 (−24.3%)	124.0 (+ 2.6%)	108.0 (+ 4.9%)	46.0 (−9.1%)	107.6 (−0.2%)	150.3 (+ 3.5%)	100.8 (+ 6.0%)
05–09	2813.0 (+ 0.1%)	1295.8 (+ 3.2%)	422.0 (+ 21.8%)	753.0 (0.0%)	360.8 (−5.4%)	513.6 (−1.5%)	112.6 (− 1.2%)	141.2 (+ 13.9%)	107.0 (−0.9%)	47.6 (+ 3.5%)	84.2 (−21.7%)	129.5 (− 13.9%)	135.5 (+ 34.5%)
10–13	2895.5 (+ 2.9%)	1271.0 (− 1.9%)	390.8 (− 7.4%)	762.8 (+ 1.3%)	404.8 (+ 12.2%)	533.5 (+ 3.9%)	118.5 (+ 5.2%)	154.8 (+ 9.6%)	108.8 (+ 1.6%)	58.3 (+ 22.4%)	71.5 (− 15.1%)	127.3 (− 1.7%)	185.1 (+ 36.5%)
Nett Change	+ 5.0%	−1.9%	+ 56.6%	−23.7%	+ 23.9%	+ 28.3%	+ 5.6%	+ 230.7%	+ 38.7%	+ 55.7%	− 30.0%	− 15.1%	+ 175.0%

Note: The change from one 5-year block to another is expressed as percentage in bracket. Nett change equals the change in value divided by the absolute value (for 2010–2013) of the original value (for 1980–1984), multiplied by 100

#### Fruits and vegetable supply

Across the years in review, there is an observed decline in the reported supply of fruit and a twofold increase in vegetable supply (Table 1). When combined as the total fruit and vegetable supply, the per capita serving size increased from 2.7 to 3.9 servings per day, assuming 80 g of fruit and vegetables per serving. It is worth noting that the FBS does not capture fruit and vegetables that are home produced, hence, it is possible that there is an underrepresentation of total fruit and vegetable supply, especially when the country was less urbanised in the 1980’s. As the country becomes more urbanised, it is no longer viable for urban dwellers to plant fruit trees and vegetables for subsistence use. As such, the observed decline in fruit supply could be steeper, and the increment of vegetables less pronounced than that observed in the FBS review.

#### Protein supply

**Fig. 2:**
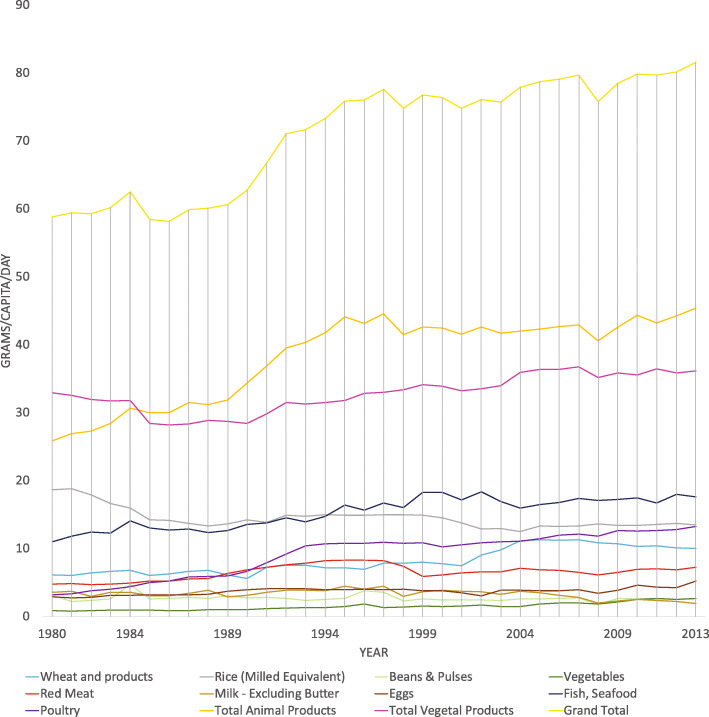
Daily supply of protein (g/capita/day) from animal and vegetal sources over the 34-year period (Source: Adapted from [13])

Overall, per capita protein supply increased by 33.8% (Table 2) during the 1980–2013 period. Initially, vegetable-sourced protein outweighed animal protein (32.2 v’s 27.8 kg/capita/day). But by 1990 the per capita supply of animal-based protein had overtaken vegetable protein. The supply of animal-based protein continued to increase at a faster rate than vegetable-sourced protein. Over the review period, the total protein available from animal products increased by 59.1% (Fig. 2) while vegetable protein increased by 11.9% (Table 2). Rice has been the predominant staple food of Malaysians and was the leading source of protein in the diet until the mid-1990s (Fig. 2). Fish succeeded rice and emerged as the top protein source since then. The trends in availability indicate that poultry supply is increasing at a rapid rate and is projected to overtake rice very soon (Fig. 2). Besides rice, the supply of wheat, an alternative source of vegetable protein, has been gradually increasing over the past three decades (Fig. 2). While fish remain the biggest contributor of animal protein supply, the importance of poultry has increased over the years (Fig. 2). Poultry emerged as the second largest animal protein contributor in 2010–2013, with a two-fold increase from 13.4% in 1980–1984 to 29.0% in 2010–2013. At the same time, the availability of protein from dairy sources declined from 18.8 to 9.9%. Red meat, as a less popular source of animal protein, has seen fairly small marginal increases (Fig. 2). At the bottom of the list are beans, pulses and leguminous vegetables, whose protein contributions are insignificant during the period under review (Fig. 2).

**Table 2:** Daily per capita supply of protein and fat from animal and vegetal sources over the 34-year period (Source: Adapted from [13])

	Availability of protein g/capita/day			Availability of fat g/capita/day		
Time period (years)	Grand Total	Vegetal Products	Animal Products	Grand Total	Vegetal Products	Animal Products
80–84	60.0	32.2	27.8	84.9	58.5	26.3
85–89	59.4 (−1.0%)	28.5 (− 11.5%)	30.9 (+ 11.0%)	90.1 (+ 6.2%)	62.5 (+ 6.8%)	27.6 (+ 4.8%)
90–94	69.1 (+ 16.3%)	30.5 (+ 7.0%)	38.6 (+ 24.8%)	87.5 (− 2.9%)	53.3 (− 14.6%)	34.1 (+ 23.6%)
95–99	76.2 (+ 10.3%)	33.0 (+ 8.2%)	43.2 (+ 11.9%)	85.6 (−2.1%)	50.8 (− 4.7%)	34.8 (+ 1.9%)
00–04	76.2 (0.0%)	34.1 (+ 3.3%)	42.1 (−2.6%)	86.4 (+ 0.9%)	55.6 (+ 9.5%)	30.8 (− 11.5%)
05–09	78.4 (+ 2.9%)	36.1 (+ 5.9%)	42.2 (+ 0.4%)	84.2 (− 2. 6%)	53.1 (− 4.6%)	31.1 (+ 1.2%)
10–13	80.3 (+ 2.5%)	36.0 (+ − 0.3%)	44.3 (+ 4.9%)	88.8 (+ 5.5%)	55.7 (+ 5.0%)	33.1 (+ 6.3%)
Nett Change	+ 33.8%	+ 11.9%	+ 59.1%	+ 4.6%	−4.8%	+ 25.6%

Note: The change from one 5-year block to another is expressed as percentage in bracket. Nett change equals the change in value divided by the absolute value (for 2010–2013) of the original value (for 1980–1984), multiplied by 100

#### Fat supply

The total fat supply has been stable over time, with only a minor increase in per capita availability (from 84.9 to 88.8 g per capita per day). Of the total fat available, in the 1980’s, around 70% was of vegetable origin (58.5 g/capita/day) and the remainder (26.3 g/capita/day) from animal sourced foods. By 2014, the balance had shifted slightly towards a higher percentage of animal sourced fat (37%), but with the majority (63%) being sourced from vegetable products (Table 2).

### Food habit pattern (MANS 2003 and 2014)

Both surveys show that the most widely consumed foods (by frequency of daily consumption) are white rice and table sugar. Between MANS 2003 and 2014, there is a decreasing trend in prevalence of consumption of rice and table sugar, which is offset by an increase in bread, wheat- and rice- based noodles, and sweetened “condensed milk”. The reduction in prevalence and frequency of daily consumption of rice was more prominent in the urban than rural populations. There is an increase in the frequency and prevalence of consumption of chicken and eggs in 2014 compared to 2003 and especially so in the urban population. While fish remains the most widely consumed animal protein, its frequency of daily consumption has decreased. Both surveys found that vegetables are more frequently and widely consumed than fruits although the frequency of daily consumption for both vegetables (< 40%) and fruits (< 7.5%) is far lower than desired. A marginal increasing trend is observed for vegetables from 2003 to 2014, while a decrease is observed in fruits.

MANS 2014 reported that 32% adults consume four heavy meals a day; the usual breakfast, lunch and dinner plus a late-night meal. However, meal pattern was not assessed nor reported in MANS 2003 so it is not possible to comment on any changes in behaviour of consumption patterns between the two data sets. Both surveys reported that milk and dairy products are not widely consumed by Malaysians. The trends observed over time in the MANS reports are consistent with those from the FBS review and those reported elsewhere [39, 42].

### Prevalence of selected NCD and NCD risk factors (NHMS I-V and MANS 2003 and 2014)

From 1996 to 2014 there was an increasing prevalence of overweight Malaysian adults (Table 3). From 2011 to 2015 the percentage remained static at around 30%, but still accounted for one third of the adult population. The prevalence of obesity showed a similar upwards trend, with an alarming increase from 4.4 to 30.6% of the adult population over a 30-year period (Table 3). Data in Table 3 may be skewed since two different cut off points for obesity were used in the different studies. Since 2011, the CPG 2004 classification was adopted for NHMS. This is based on the WHO [60] recommendation that the CPG criteria are used for Asians since they tend to have higher amounts of abdominal fat at lower BMIs [60] which poses a higher risk for obesity-related morbidity and mortality. The use of the reduced BMI threshold level for obesity may have skewed the magnitude of prevalence, but was not formally discussed in the respective NHMS reports. Using the new classification, about two-third of the adult population was overweight or obese in 2015 (Table 3). On the other hand, the prevalence of underweight among adults above 18 years old has fallen to 6.2% in NHMS 2015 from 25.2% previously reported in NHMS 1996. It is not obvious whether this is an anomaly due to the BMI cut-off points utilised, or if it is a reflection of the increasing body size of Malaysians.

**Table 3:** The prevalence of overweight and obesity in adults of 18 years and above (Source: data for NHMS III (2006), NHMS IV (2011) and NHMS V (2015) from Institute of Public Health [21, 28], for NHMS I (1986) and II (1996) from Ahmad [2], and for MANS (2003) and MANS (2014) from Institute of Public Health [24, 30])

	NHMSII (1996) %	MANS (2003) %	NHMSIII (2006) %	NHMS IV (2011) %	MANS (2014) %	NHMS V (2015) %
Overweight	16.6^a^	26.7 ^a^	29.1 ^a^	33.3 ^b^	32.4 ^a^	33.4 ^b^
Obese	4.4 ^a^	12.2 ^a^	14.0 ^a^	27.2 ^b^	18.5 ^a^	30.6 ^b^

^a^ According to WHO classification, overweight is defined as BMI ≥ 25 kg/m2 and < 30 kg/m2 and obesity as BMI ≥ 30 kg/m2.

^b^ According to CPG (2004) classification, overweight is defined as BMI ≥ 23.0 kg/m2 and < 27.4 kg/m2 and obesity as BMI ≥ 27.5 kg/m2.

Evaluation of NHMS reports across the years highlighted an increasing prevalence (170% increase) in diabetes mellitus from 8.3% in 1996 to 22.5% two decades later in 2015 (Table 4). Data from sequential NHMS show a rapid doubling (over 200%) in the prevalence of hypertension from 1986 to 2006, which has remained fairly constant, at about 40% of adults over 30 years of age, since 2006 (Table 4). In line with the incidence of other NCD, the prevalence of hypercholesterolaemia was high and on the increase (Table 4). About half of the adult population had high blood cholesterol. Prevalence among adults aged 30 years and above had increased more than fourfold from 11.7% in 1996 to 56.8% in 2015 (Table 4).

**Table 4:** Prevalence of NCD in adults of 30 years old and above (Source: data for NHMS III (2006), NHMS IV (2011) and NHMS V (2015) from Institute of Public Health [20, 22, 28] and for NHMS I (1986) and II (1996) from Ahmad [2])

	NHMS I (1986) %	NHMS II (1996) %	NHMS III (2006) %	NHMS IV (2011) %	NHMS V (2015) %
Total Diabetes	6.3^a^	8.3	14.9	20.8	22.5
Total Hypertension	14.4^b^	32.9	42.6	43.5	39.8
Total Hypercholesterolaemia	n/a	11.7	28.2	43.9	56.8

Note: Prevalences are for adults of 30 years old and above except for “a” (≥ 35 yrs) and “b” (≥25 yrs)

The results are in keeping with the epidemiological transition that occurs in nations with rapidly evolving economies, and with the nutrition transition from receding famine to increasing prevalence of degenerative disease [44]. It could also be suggestive of a low awareness of health screening among Malaysian. Of particular concern is the increase in the number of undiagnosed NCD cases (Table 5). More than half of adults above 18 years old with diabetes and hypercholesterolaemia in Malaysia were not aware of their condition until they were tested during NHMS sampling. Although the prevalence of undiagnosed hypertension is high, there has been a fall in prevalence between 2006 and 2015.

**Table 5:** Prevalence of undiagnosed NCD in adults of 18 years old and above (Source: data for NHMS III (2006), NHMS IV (2011) and NHMS V (2015) from Institute of Public Health [20, 22, 28] and for NHMS I (1986) and II (1996) from Ahmad [2])

	NHMS I (1986) %	NHMS II (1996) %	NHMS III (2006) %	NHMS IV (2011) %	NHMS V (2015) %
Undiagnosed Diabetes ^*^	1.8^a^	1.8^b^	4.5	8.0	9.1
Undiagnosed Hypertension^**^	n/a	n/a	23.9	19.8	17.2
Undiagnosed Hypercholesterolaemia^***^	n/a	n/a	18.4	26.6	38.5

Note: Prevalences are for adults of 18 years old and above except for “a” (≥ 35 yrs) and “b” (≥30 yrs); * respondent was not known to have diabetes and had a fasting capillary blood glucose (FBG) of ≥6.1 mmol/L or more (or non-fasting blood glucose of ≥11.1 mmol/L); ** respondent was not known to have hypertension and had a systolic blood pressure of ≥140 mmHg and/or diastolic blood pressure of ≥90 mmHg; *** respondent was not known to have hypercholesterolaemia and had a total blood cholesterol of ≥5.2 mmol/L

## Discussion

It is evident from the preceding data that Malaysia is a nation in the midst of transition. The demographic and epidemiological transitions that have taken place over the 34 year period in review have the hallmark of transitions taking place in other rapidly evolving economies. Currently, there is very little sign that this is going to change. The prevalence of obesity and associated NCD has continued to increase amongst Malaysians, frequently surpassing the global average (Prevalence of Selected NCD and NCD Risk Factors (NHMS I-V and MANS 2003 and 2014)). In comparison to the WHO 2014 global average, diabetes mellitus was three times more prevalent in Malaysia [62]. While the prevalence of overweight was on par with the [62] global average, obesity was about 2.5 times more prevalent in Malaysia [64]. Hypercholesterolaemia was also approximately 50% more prevalent in Malaysia compared to the WHO 2008 global average [63]. Since 2014, Malaysia has topped the league tables as the most obese nation in South East Asia [40].

The next section of the discussion examines the underlying causes of the nutrition transition in Malaysia. It is divided into three main areas: The food environment; Changes in lifestyle and behaviour; Government policies, including taxes and subsidies. After examining the underlying factors, recommendations are made for policy changes to address the issues and provide better health outcomes for Malaysians.

### The food environment

#### An abundance of food

A decreasing rate of population growth (Transitioning Demographic (DoSM)) in combination with a strong growth in GDP (Rapid Economic Growth and Transition (DoSM)) has increased access to and availability of more and cheaper food, also known as the “expansion” stage of nutrition transition [34]. For the period under review, there has been an abundance of calories available, indicating that Malaysia has been in the expansion stage of transition for over 35 years. Between 2010 and 2013, the average Malaysian woman and man had access to around 93 and 45% more calories than needed (Changes in Trends of Energy and Nutrient Supply (FBS Review)). Vandevijvere et al. [54] warned that the obesity epidemic in around 80% of countries surveyed is fuelled by the sheer abundance of food as it was found that an increase in the amount of per-capita food energy available has created excesses that alone can account for increases in average body weight. Only countries hit by famine, natural disasters or civil war did not follow the trend [54]. The ever-increasing prevalence of obesity and persistent surplus of per capita food energy supply in Malaysia is consistent with the findings of Vandevijvere et al. [54].

#### Change in types of foods consumed

During the period under review, Malaysia entered the ‘substitution phase’ of the nutrition transition. This phase is typified by a shift in the types of food consumed with no major change in the overall energy supply [34]. The shifts in food groups are usually described as an increase in consumption of refined carbohydrates, added sweeteners, edible oils, and animal-sourced foods, together with a reduction in legumes, other vegetables and fruits [47]. This transition may differ from one country to another due to differences in culture, beliefs and religious traditions [34].

In Malaysia, there are several reasons for the preference for chicken and fish over red meat, not least the price, but also the convenience, versatility, ready availability and acceptability to all ethnicities. Malaysia is a multiracial and multi-religion nation, in which all populations accept fish, seafood and chicken compared to red meat, such as pork and beef that are prohibited among the Muslims and Hindus, respectively. It is also a norm among Chinese who adopt some Buddhism beliefs to avoid beef. The Malaysian climate and topography (no grassland) is not suited to the rearing of beef or dairy cattle. Poultry farming, for meat and egg production takes place on an intensive scale, making chicken meat and eggs available at very attractive prices. With regards to fish consumption, Malaysia is an island nation, thus it makes sense that fish are the traditional form of animal protein. Locally caught or farmed fish are cheap and plentiful.

Modern healthy eating advice has advocated the consumption of lean white meat (chicken) over red meat, which is perceived to contain a higher concentration of saturated fats. While this may have been the case 60–70 years ago, the modern chicken, fast growing and intensively reared, has a very different nutrient composition profile to its ancestors. They provide more energy from fat than protein and contain more than twice as much fat as it did in 1940, a third more calories and a third less protein [57]. The method of cooking also has a significant impact on the nutritional value of the meat consumed. Deep fried chicken tastes better than boiled or steamed chicken, and is a favourite snack and meal. Not only chicken that is deep fried, the most preferred fish cooking style by Malaysians is deep-frying [4]. During frying, fish will absorb some of the oil, increasing its calorie content and changing the types of fat it contains, with the amount of beneficial fatty acids particularly adversely affected.

Another trend also common in transitioning countries is the increase in wheat consumption [43, 45, 49]. Malaysia too is consuming more wheat at the expense of rice. It is important to note that wheat is usually consumed in its highly processed form of flour, whereas rice is traditionally polished and boiled or steamed before consumption. Different varieties of wheat are imported and refined to produce noodles, cakes, cookies, crackers, buns and breads – all of which are commodities that have roles to play as convenience foods. To put things into perspective: a bowl of steamed rice is less processed and less “value-added” than any one of the wheat-based products available in Malaysia. Wheat-based foods are also consumed in a more calorific but less nutritious way. For instance, the ubiquitous breakfast item, “Roti Canai” or Malaysian flatbread, is made from refined-wheat-flour dough which is repeatedly kneaded, flattened, oiled, and folded before proofing and shallow frying in more oil. In addition, white toast is not complete without margarine spread. Coincidentally or not, partially hydrogenated oil that is linked to the development of NCD is one common vital ingredient in the aforementioned wheat-based products. This industrial source of trans-fat is not banned in Malaysia, unlike most high income countries that have heeded WHO’s call on eliminating it in our diets by 2023.

While other studies of the nutrition transition in developing economies [43, 45, 49] found an increase in milk intake, the opposite holds true in Malaysia. Per capita milk supply has reduced from two-third to half a glass a day. Akin to cattle rearing, dairy farming is equally not suited to the Malaysian climate, hence there has been no culture of milk production or consumption. Whilst there is some locally produced fresh milk, the majority of milk and dairy products are made from imported milk powder and hence are not affordable by all [51]. Additionally, the rate of lactose intolerance among adults in Malaysia is extremely high at over 80% [6, 16], therefore most adults choose to avoid milk and dairy products. Malaysian adults tend to substitute milk in beverages such as tea and coffee with sweetened “condensed milk” that is easier on the palate, stomach and pocket but is poorer for health. It is important to highlight that the ubiquitous sweetened “condensed milk” widely consumed in Malaysia is actually the non-dairy creamer, made from palm oil derivatives. The dairy version of condensed milk is less widely available and usually imported to cater for niche upmarket consumers.

The FBS highlighted an over-abundance of and sustained increase in the supply of sugar and sweeteners over the past three decades (Changes in Trends of Energy and Nutrient Supply (FBS Review)). While the recommended upper daily limit for sugar is no more than 9 or 6 teaspoons a day, men and women respectively, the amounts available in the 1980s were equivalent to 21 teaspoons per person per day. The availability of sugar and sweeteners has continued to increase over the decades, hand in hand with the increase in prevalence of NCD and obesity.

### Changes in lifestyle and behaviour

In keeping with Popkin’s findings (2015), underlying the afore discussed transition in Malaysia are the growth of the modern retail culture, a change in technology affecting physical activity and inactivity, access to mass media, urbanisation and penetration of modern food systems into all societies. The huge demographic change in Malaysia, from a nation that was largely rural (72%) in the 1960’s to a complete reversal of this situation by 2014, brought with it concomitant changes in lifestyle, behaviour and eating habit. It is difficult to disaggregate the individual factors from each other as one change has knock on effects on other factors. Together they fall under the banner of urbanisation. They have a significant impact on the eating behaviour and health outcomes of the population.

#### Sedentary urban living

Over three quarters of Malaysians now live in urban or peri-urban areas. They are linked by fast highways, by high speed internet and have access to technologies that are designed to make their lives more comfortable and convenient. Less energy is expended both at work and in traveling to work; people have sedentary occupations and tend to walk less as car ownership continues to rise. Even if one’s energy intake stays the same over the years, the gradual metabolic ageing causing reduction in basal energy requirement and reduction in energy expenditure due to sedentary lifestyle can bring about energy surplus that leads to a gradual, but persistent, weight gain over a considerable period of time.

#### Eating out

Changing lifestyles, mainly due to work commitment, have fuelled the increase in numbers eating out and the need for convenience foods. According to the Malaysian Food Barometer, more than 64% of Malaysians eat at least one meal per day outside of home; of the remaining 36% who eat at home, 12.5% have at least one meal that comes from outside [48]. Traditionally, foods were home cooked local dishes prepared by mothers. Due to an increasing number of “dual-income” families, eating out becomes increasingly prevalent in Malaysia. It is also not surprising that the review has found that about one-third of the population consume a fourth heavy meal late at night. In fact, around-the-clock dining has become so inherent with Malaysian culture that the government had to retreat a proposal to ban eateries from operating 24 h after facing community backlash [17].

The abundance of eateries in Malaysia makes eating out an easy option. Consumers do have the choice to select home-cooked fare such as the “nasi kandar” or “nasi campur” stalls that offers an abundance of local dishes - vegetables, fish, chicken, and curries of various kinds - served with limitless steamed rice. These eateries do provide healthy and less healthy options. It is left to the consumer to make choices and to select the appropriate amount to consume. In addition to the “healthy” choices, an abundance of small hawker stalls offer a range of food choices for a very reasonable price. In general, these foods tend to be fried, high in salt, sugar and devoid of nutrients. The availability of “ready meals” in convenience stores has mushroomed over the past 5 years. These ready meals do provide a safety net for those who do not have access to food storage or preparation facilities, but can be viewed as one of the contributory factors to lifestyle and dietary change and associated health outcomes.

#### Trendy food culture

The establishment of shopping malls in all towns has contributed to the globalisation of food products and culture that is further promoted and instilled in the local communities by their increasing presence across all towns in Malaysia, big and small. Outlets serving trending food is fast becoming a quintessential component of the evolving urban food culture. The long queue in front of these outlets cannot be missed. The latest trend in Asia, and in Malaysia, is the consumption of “bubble tea”, a high calorie sweetened tea-flavoured beverage with chewy tapioca balls. Coffee culture is also on the increase in Malaysia. The trending coffee shops are the well-known global brands, reinforced by independent local businesses that serve large volumes of sweetened, milk based beverages and cakes and pastries alongside. Kiosk chains market juice and smoothie drinks as healthy and offering a range of purported benefits such as “immune-boosting” and “cleansing”. All are sold in large volumes and, as with the bubble tea and coffee, some of these products are more akin to a meal than a beverage in terms of the total calorie count. For instance, a search on the webpage of one of the well-known retailers found that one of their popular products marketed as a protein drink provides 588.5 cal. A single serving of a “bubble tea” contains 299 cal and 38 g of sugar [37].

#### The internet era

The majority of Malaysians (75% in 2015) now have access to the internet. Whilst this service has numerous positive attributes –access to information being one of these – it also has negative attributes with respect to food access and availability. Improved internet access enables the online ordering of food (24 h a day), which can be home delivered. This further reduces the necessity to expend energy while searching for the next meal or snack. While there is a lack of official statistics from the Government, it has been reported elsewhere that 80% of the population is active social media user spending about 2.8 h per day on average just on social media [36]. The Instagram age of sharing trending foods with friends means that eating out is rapidly evolving to become more of a social pastime and hobby. It gives individuals kudos and bragging rights over their friends and peers. Eating out, especially at the latest trending place, is aspirational and seen as a form of success.

### Government policies

The epidemiological transition in Malaysia is reflected in the parallels between evolving economies and a growing burden of NCD. Being aware of this situation, the government has implemented since 1996 national plans of actions in an attempt to curb it.

#### Nutrition action plans and policies to improve health

Since the early 1990s, Government efforts have focused on the coordination of nutrition intervention programmes in the country between various agencies [52]. The first blueprint was the National Plan of Action for Nutrition in Malaysia (NPANM I) (1996 to 2000). This plan was primarily targeted at undernutrition. Meanwhile, the problem with overweight was recognised for the first time and strategies to control unhealthy weight gain in the population were outlined in NPANM I. Unfortunately, not only did the strategies fail to have any impact, the prevalence of overweight and obesity rose by 60 and 177% respectively during the period of NPANM I implementation. At the same time, the incidences of diet-related NCD continued to rise at an alarming rate. It was not until the following NPANM II (2006–2015) and NPANM III (2016–2025) that the needs to prevent and control diet-related NCD were recognised. According to the latest NHMS, strategies to reduce diet-related NCD are underwhelming in effect as the prevalence of NCD has continued to rise over the years, with some figures surpassing the global average.

It is important to understand why these national plans of action were ineffective. Perhaps one of the contributing factors is that the strategies to promote healthy eating and active living over the years used a downstream marketing approach. The fact that the upstream factor of an abundance of cheap, readily available calories can cause problems were overlooked in all these policies. Policymakers expected a trickle down approach, of advertisements and handouts that implored people to eat less and do more exercise, to be whole heartedly adopted by the general population. While at the same time, the population was embracing the rapid development that urbanisation and globalisation brought with it. Requesting people to change or moderate their behaviour while living in a state of abundance, is difficult to achieve. Behavioural interventions have been deemed an ineffective means to prevent obesity in both adults and children mainly due to strong influences beyond individual control [11]. However, to succeed, this method requires more stringent measures to enable people to make healthy choices.

#### Subsidies and taxes

Whilst the MoH devised policies aimed at improving health outcomes, other sectors of the Government offered a range of subsidies and incentives to ensure ‘cheap food for all’. Annual Government spending on subsidising basic necessities is at least RM2 billion (USD 450 million) [35]. This allocation includes agricultural subsidies, especially rice production incentives, while part of the allocation is also spent on consumer subsidies. The four subsidised consumer items are sugar, flour, rice and cooking oil, all of which are energy dense, nutrient poor foods. This subsidy could partially explain the increasing per capita supply and surplus of such products. The abundance of excess calories has been exacerbated by the introduction of policies to subsidise these nutrient-poor, energy dense food items. Continued subsidy to increase access to and availability of these foods is paradoxical.

### Ways forward

The kind of food that is most available, affordable and convenient is a major determinant of people’s diets [14]. According to this review, most of the Malaysian population failed to consume adequate amounts of fruit and vegetable (Changes in Trends of Energy and Nutrient Supply (FBS Review)). This is not surprising since the local supply has never been enough to support the recommended intake of at least 5 servings per person per day. The Malaysian fruit and vegetable industry is generally neglected. The emphasis of agricultural development has been on rice and the commodity crops of oil palm, rubber, cocoa and recently durian [8, 9]. To increase accessibility, policymakers need to offer at least as much research and financial support to local fruits and vegetable farmers. Subsidies strike the best balance between effectiveness in changing behaviours and long-term monetary benefits to society [15]. Instead of subsidising the aforementioned energy dense foods, fruits and vegetables should be subsidised as an incentive to encourage healthier eating habits. Reduced fruit and vegetable consumption is often linked to poor health and increased risk of NCD. The protective effects of fruits and vegetables in the diet may be due to their high content of micronutrients and fibre, acting through mechanisms such as lowering blood pressure [1, 50, 59], improving lipoprotein profile [19] and increasing insulin sensitivity [5, 58].

In tandem with subsidies for fruits and vegetables, fiscal disincentives can be useful for creating consumer demand for nutritious foods [14]. By exerting pressure on the food industry to improve food environments, food taxes such as the sugar tax can be effective at changing people’s diet. From July 2019, the Ministry of Finance (MoF) introduced a sugar tax levy akin to Mexico’s on the manufacturer of canned/bottled sugar-sweetened beverages, i.e. non-alcoholic beverages containing added sugars of more than 5 g per 100 ml drink and fruit or vegetable juice drink containing added sugars of more than 12 g per 100 ml drink. The sugar tax is very much in its infancy and it is impossible to tell whether it is likely to succeed in Malaysia in the absence of longitudinal epidemiological data. At a glance, the directive as it stands is unlikely to succeed, mainly because it is failing to address the real sugar problem – the consumption of sugar from sources other than canned/bottled drinks. Table sugar and non-dairy sweetener, as implied in the findings of MANS 2003 and 2014 conducted by the MoH, are not included in the proposed sugar tax. The most frequently consumed beverages are those prepared at home or at eateries, with added table sugar and/or non-dairy sweetener. A survey by Ahmad in 2015 reported that about 98.6% adults consumed on average two glasses of these beverages per day [3].

The rapid change in Malaysia’s food environment on top of the persistent overabundance of energy supply in the country has tipped the scales in favour of NCD proliferation. Policymakers have mostly overlooked the upstream links between poor diet and policy determinants of food supply from farm to shelves [56] and the importance of employing multicomponent, multidisciplinary and multifaceted approaches for obesity management [7]. As it has been in the past until now, the burden to reduce malnutrition and NCD lies mainly with the MoH. There is a need for setting an overarching vision and common goals for policymakers from different Ministries to meet for achieving the vision with a supportive policy ecosystem to facilitate that. In addition to the MoH, other Ministries with relevant roles to play include the Ministries of Agriculture, Domestic Trade and Consumer Affairs, International Trade and Industry, Education, Finance and Woman and Family. Coherence between different ministerial policies to promote healthy food environments should be ensured and progress towards achieving the goals should be measurable. A good case in point is the MoF and its sugar tax. How will the effectiveness of the sugar tax be measured? If the Key Performance Indicators for the MoF is only of balanced budget and fiscal sustainability, where does its accountability for obesity sit? Would the sugar tax be more robust had the MoF worked closer with other Ministries?

## Conclusion

The quasi-historical examination of nutrition and health scenario in Malaysia reveals that past interventions have largely been ineffective to curb the growing obesity and NCD epidemic. As Malaysia proceeds rapidly towards a developed economy status, the population’s lifestyle will continue to change. Evidences point to the fact that the local food supply and environments have become increasingly obesogenic. Policymakers may have to learn from post-mortems of past policies, re-examine its present policies and strategies, and formulate sustainable, comprehensive and multifaceted actions to ensure a conducive, healthy and nutritious food systems and environment for its population. The epidemic is a complex issue and requires strong political will and concerted effort from all stakeholders.

## Data Availability

Data sharing is not applicable to this article as no datasets were generated during the current study. The datasets analysed during the current study are drawn from external open-portal data sources as described in the Method section and their respective permalinks are listed in the Reference section.

## Abbreviations

BMI: Body mass index
CPG: Clinical Practice Guidelines
FAO: Food and Agriculture Organisation of the United Nations
FAOSTAT: Food and Agriculture Organization Corporate Statistical Database
FBS: Food balance sheet
FFQ: Food frequency questionnaire
FV: Fruits and vegetables
GDP: Gross domestic product
MANS: Malaysian Adult Nutrition Survey
MoF: Ministry of Finance
MoH: Ministry of Health
NCD: Non-communicable diseases
NHMS: National Health and Morbidity Surveys
NPANM: National Plan of Action for Nutrition in Malaysia
WHO: World Health Organisation
RNI: Recommended nutrient intakes

## References

[1] Aburto NJ, Hanson S, Gutierrez H, Hooper L, Elliott P, Cappuccio FP (2013). Effect of increased potassium intake on cardiovascular risk factors and disease: systematic review and meta-analyses. BMJ.

[2] Ahmad, A. J. (2011). Reorient Health Services [PowersPoint slides]. Retrieved from www.infosihat.gov.my/infosihat/isusemasa/power%20point/ReorientHealthSvcs.pptx.

[3] Ahmad MH. Food Consumption Patterns: Findings from the Malaysian Adults Nutrition Survey (MANS) 2014. Med J Malays. 2015;70.

[4] Ahmad NI, Wan Mahiyuddin WR, Tengku Mohamad TR, Ling CY, Daud SF, Hussein NC (2016). Fish consumption pattern among adults of different ethnics in peninsular Malaysia. Food Nutrition Ress.

[5] Bazzano LA (2005). Dietary intake of fruit and vegetables and risk of diabetes mellitus and cardiovascular diseases (pp. 66–66).

[6] Asmawi MZ, Seppo L, Vapaatalo H, Korpela R (2006). Hypolactasia & lactose intolerance among three ethnic groups in Malaysia. Indian J Med Res.

[7] Cochrane AJ, Dick B, King NA, Hills AP, Kavanagh DJ (2017). Developing dimensions for a multicomponent multidisciplinary approach to obesity management: a qualitative study. BMC Public Health.

[8] Dardak, R. A. (2015). Transformation of agricultural sector in Malaysia through agricultural policy. Food and fertilizer technology center-agricultural policy platform (FFTC-AP). Food and fertilizer Technology Center for the Asian and Pacific Region. Malaysian agricultural Research and Development Institute (MARDI), Malaysia. Retrieved from http://ap.fftc.agnet.org/ap_db.php?id=386.

[9] Dardak, R. A. (2019). Trends in production, trade and consumption of tropical fruit in Malaysia. Food and fertilizer technology center-agricultural policy platform (FFTC-AP). Food and fertilizer Technology Center for the Asian and Pacific Region. Malaysian agricultural Research and Development Institute (MARDI), Malaysia. Retrieved from: http://ap.fftc.agnet.org/ap_db.php?id=1015.

[10] DoSM (2016). National demographics and socio-economic data were obtained from official spreadsheets and reports uploaded by the Department of Statistics, Malaysia on its open data portal available at.https://www.dosm.gov.my/v1/index.php?r=column3/accordion&menu_id=aHhRYUpWS3B4VXlYaVBOeUF0WFpWUT09#.

[11] Elinder LS (2005). Obesity, hunger, and agriculture: the damaging role of subsidies. BMJ.

[12] FAO (2001). FAO food balance sheets: a handbook.

[13] FAO (2017). Food Balance Sheets for year 1980 to 2013 were downloaded as spreadsheets available at http://faostat.fao.org/site/368/default.aspx#ancor.

[14] FAO/WHO (2018). Proceedings of the FAO/WHO international symposium on sustainable food systems for healthy diets and improved nutrition. Rome.

[15] Flores M, Rivas J (2017). Cash incentives and unhealthy food consumption. Bull Econ Res.

[16] Goh LH, Mohd Said R, Goh KL (2018). Lactase deficiency and lactose intolerance in a multiracial Asian population in Malaysia. JGH Open.

[17] Hamdan, N. (2015). Najib: no ban on 24-hour eateries. The star. Retrieved from https://www.thestar.com.my/News/Nation/2015/06/27/Najib-no-ban-24-hr-makan/.

[18] Hawkes C, Chopra M, Friel S. Globalization, trade, and the nutrition transition. In: Globalization and health. Routledge; 2009. p. 257–84.

[19] Heiss C, Keen CL, Kelm M (2010). Flavanols and cardiovascular disease prevention. Eur Heart J.

[20] Institute of Public Health (2006a). National Health and morbidity survey 2006 (NHMS III). Diabetes Mellitus. Downloaded from:http://iku.moh.gov.my/images/IKU/Document/REPORT/2006/DiabetesMellitus.pdf.

[21] Institute of Public Health (2006). National Health and morbidity survey 2006 (NHMS III).

[22] Institute of Public Health (2006). National Health and morbidity survey 2006 (NHMS III).

[23] Institute of Public Health (2008a). Malaysian Adult Nutrition Survey (MANS) 2003 Vol. 2: General Findings. Downloaded from:http://iku.moh.gov.my/images/IKU/Document/REPORT/MANS2003/Volume2-GeneralFindings.pdf.

[24] Institute of Public Health (2008b). Malaysian Adult Nutrition Survey (MANS) 2003 Vol. 3: Nutritional Status of Adults Aged 18 to 59 Years. Downloaded from:http://iku.moh.gov.my/images/IKU/Document/REPORT/MANS2003/Volume3-NutritionalStatus.pdf.

[25] Institute of Public Health (2008c). Malaysian Adult Nutrition Survey (MANS) 2003 Vol. 4: Meal Pattern of Adults Aged 18 to 59 Years. Downloaded from:http://iku.moh.gov.my/images/IKU/Document/REPORT/MANS2003/Volume4-mealpattern.pdf.

[26] Institute of Public Health (2008d). Malaysian Adult Nutrition Survey (MANS) 2003 Vol. 5: Dietary Intake of Adults Aged 18 to 59 Years. Downloaded from: http://iku.moh.gov.my/images/IKU/Document/REPORT/MANS2003/Volume5-DietaryIntakeOfAdults.pdf.

[27] Institute of Public Health (2008e). Malaysian Adult Nutrition Survey (MANS) 2003 Vol. 7: Habitual Food Intake of Adults Aged 18 to 59 Years. Downloaded from:http://iku.moh.gov.my/images/IKU/Document/REPORT/MANS2003/Volume7-HabitualFoodIntake.pdf.

[28] Institute of Public Health (2011). National Health and morbidity survey 2011 (NHMS IV). Vol. II: NonCommunicable Diseases. Downloaded from:http://iku.moh.gov.my/images/IKU/Document/REPORT/NHMS2011-VolumeII.pdf.

[29] Institute of Public Health (2014a). Malaysian Adult Nutrition Survey (MANS) 2014 Vol. I: Methodology and General Findings. Downloaded from:http://iku.moh.gov.my/images/IKU/Document/REPORT/NHMS2014-MANS-VOLUME-1-MethodologyandGeneralFind.pdf.

[30] Institute of Public Health (2014b). Malaysian adult nutrition survey (MANS) 2014 Vol. II: Survey Findings. Downloaded from:http://iku.moh.gov.my/images/IKU/Document/REPORT/NHMS2014-MANS-VOLUME-2-SurveyFindings.pdf.

[31] Institute of Public Health (2014c). Malaysian adult nutrition survey (MANS) 2014 Vol. III: Food Consumption Statistics of Malaysia. Downloaded from:http://iku.moh.gov.my/images/IKU/Document/REPORT/NHMS2014-MANS-VOLUME-3-FoodConsumptionStatisticsofMalaysia.pdf.

[32] Institute of Public Health (2015). National health and morbidity survey 2015 (NHMS V). Vol. II: Non-Communicable Diseases, Risk Factors and Other Health Problems. Downloaded from:http://iku.moh.gov.my/images/IKU/Document/REPORT/nhmsreport2015vol2.pdf.

[33] Kaur, S. (2018). 700 shopping malls by end of next year. New Strait times. Retrieved from https://www.nst.com.my/property/2018/10/424762/700-shopping-malls-end-next-year.

[34] Kearney J (2010). Food consumption trends and drivers. Philosophical Transactions of the Royal Society of London B. Biol Sci.

[35] Khairul, A. M. (2017). Domestic trade minister regrets negative spin on statements regarding rising prices of goods and services; Govt spends RM2b on basic food subsidies. New Strait times. Retrieved from https://www.nst.com.my/news/nation/2017/12/313080/domestic-trade-minister-regrets-negative-spin-statements-regarding-rising.

[36] Malaysia Digital Association (2016). Malaysia Digital Landscape: Exploring the Digital Landscape in Malaysia Boosting Growth for a Digital Economy. Retrieved from http://www.malaysiandigitalassociation.org.my/wp-content/uploads/reports/2016/mda-2016-malaysia-digital-landscape.pdf.

[37] Min JE, Green DB, Kim L (2017). Calories and sugars in boba milk tea: implications for obesity risk in Asian Pacific islanders. Food Sci Nutrition.

[38] Ministry of Health Malaysia (2017). Recommended nutrient intakes for Malaysia. A report of the technical working group on nutritional guidelines. Downloaded from http://nutrition.moh.gov.my/wp-content/uploads/2017/05/FA-Buku-RNI.pdf.

[39] Mirnalini K, Zalilah MS, Safiah MY, Tahir A, Siti HM, Siti RD (2008). Energy and nutrient intakes: findings from the Malaysian adult nutrition survey (MANS). Malays J Nutr.

[40] Ng M, Fleming T, Robinson M, Thomson B, Graetz N, Margono C (2014). Global, regional, and national prevalence of overweight and obesity in children and adults during 1980–2013: a systematic analysis for the global burden of disease study 2013. Lancet.

[41] Noor MI (2002). The nutrition and health transition in Malaysia. Public Health Nutr.

[42] Norimah AK, Safiah M, Jamal K, Haslinda S, Zuhaida H, Rohida S (2008). Food consumption patterns: findings from the Malaysian adult nutrition survey (MANS). Malays J Nutr.

[43] Pingali P (2007). Westernization of Asian diets and the transformation of food systems: implications for research and policy. Food Policy.

[44] Popkin BM, Gordon-Larsen P (2004). The nutrition transition: worldwide obesity dynamics and their determinants. Int J Obes.

[45] Popkin BM (2006). Global nutrition dynamics: the world is shifting rapidly toward a diet linked with noncommunicable diseases. Am J Clin Nutr.

[46] Popkin BM, Adair LS, Ng SW (2012). NOW AND THEN: the global nutrition transition: the pandemic of obesity in developing countries. Nutr Rev.

[47] Popkin BM (2015). Nutrition transition and the global diabetes epidemic. Current diabetes reports.

[48] Poulain JP, Tibère L, Laporte C, Mognard E (2014). Malaysian food barometer.

[49] Schmidhuber J, Shetty P (2005). The nutrition transition to 2030. Why developing countries are likely to bear the major burden. Acta Agriculturae Scand Section C.

[50] Streppel MT, Arends LR, van’t Veer P, Grobbee DE, Geleijnse JM (2005). Dietary fiber and blood pressure: a meta-analysis of randomized placebo-controlled trials. Arch Intern Med.

[51] Suntharalingam C, Kusano E (2019). Marketing mix of Milk and dairy products in peninsular Malaysia. Food Value Chain in ASEAN: Case Studies Focusing on Local Producers. ERIA.Research Project Report FY2018 no.5.

[52] Tee, E. S. (2007). Plan of action. The Star Newspaper. Retrieved from https://www.thestar.com.my/lifestyle/health/2007/07/08/plan-of-action/#acUwzQOyqZ3ZViQR.99.

[53] Tourism Malaysia (2015). Malaysia’s shopping landscape with main and new shopping precincts. Retrieved from https://www.tourism.gov.my/media/view/malaysia-s-shopping-landscape-with-main-and-new-shopping-precincts.

[54] Vandevijvere S, Chow CC, Hall KD, Umali E, Swinburn BA (2015). Increased food energy supply as a major driver of the obesity epidemic: a global analysis. Bull World Health Organ.

[55] Vepa, S. S. (2004). Impact of globalization on the food consumption of urban India. Globalization of food Systems in Developing Countries: impact on food security and nutrition, FAO food and nutrition paper 83, Food and Agriculture Organization of the United Nations, Rome. 19178111

[56] Wallinga D (2010). Agricultural policy and childhood obesity: a food systems and public health commentary. Health Aff.

[57] Wang Y, Lehane C, Ghebremeskel K, Crawford MA (2010). Modern organic and broiler chickens sold for human consumption provide more energy from fat than protein. Public Health Nutr.

[58] Weickert MO, Pfeiffer AF (2008). Metabolic effects of dietary fiber consumption and prevention of diabetes. J Nutr.

[59] Whelton SP, Hyre AD, Pedersen B, Yi Y, Whelton PK, He J (2005). Effect of dietary fiber intake on blood pressure: a meta-analysis of randomized, controlled clinical trials.

[60] WHO (2000). World Health Organization international obesity task force. The Asian-Pacific perspective: redefining obesity and its treatment.

[61] WHO (2011). International Statistical Classification of Diseases and Related Health Problems, 10th Revision: Instruction Manual, vol. 2. 2011.

[62] WHO (2016). Global report on diabetes.

[63] WHO (2018). Obesity and overweight. fact sheets.

[64] WHO (n.d.). Raised Cholesterol: Situation and trends. Global Health Observatory (GHO) data. Retrieved from https://www.who.int/gho/ncd/risk_factors/cholesterol_text/en/.

